# Peripartum Cardiomyopathy and Takotsubo Cardiomyopathy: Rare Contributors to Cardiac Causes of Maternal Mortality

**DOI:** 10.7759/cureus.76621

**Published:** 2024-12-30

**Authors:** Taylor A LaValle, Rebecka M Ernst, Ahmed Robbie

**Affiliations:** 1 Neurology, Mercy Hospital Springfield, Springfield, USA

**Keywords:** brain death, cardiac arrest, cardiomyopathy, maternal mortality, peripartum

## Abstract

Peripartum cardiomyopathy (PPCM) and takotsubo cardiomyopathy (TCM) are cardiac conditions that can occur in the peripartum period. They have distinct characteristics and incidence rates; although rare, both contribute to the second leading cause of all-cause maternal mortality in the state of Missouri. PPCM can lead to heart failure, and TCM can cause acute arrhythmias leading to sudden cardiac death in otherwise healthy individuals. Our patient developed PPCM and TCM, which led to myocardial infarction, resulting in anoxic brain injury at 10 months postpartum. This case presentation demonstrates the need to consider pregnancy-related conditions up to 12 months postpartum. We postulate that the cardiomyopathies could have presented as early as five to eight weeks postpartum. Had our patient received screening for cardiac disease, resulting in earlier diagnosis and treatment, she may still be alive today. Increased surveillance and suspicion of cardiac diseases are crucial to reducing early and late postpartum mortality. Our patient presented to the ER for a headache three days before developing cardiac arrest. Our recommendation would be to screen any asymptomatic woman up to 12 months postpartum with an electrocardiogram and brain natriuretic peptide and compare the results to the baseline, if available. If any results from the screening tests are abnormal, clinicians should further investigate with troponin levels and echocardiography. We recommended reporting this case to the Missouri Maternal Mortality Review Committee and a hospital autopsy be performed for further investigation and contribute to our understanding of high maternal deaths in the state.

## Introduction

Maternal mortality is about 20 deaths per 100,000 live births in the United States, which has returned to baseline after increasing during the 2020-2022 COVID-19 pandemic. In the state of Missouri, the leading cause of pregnancy-related deaths is cardiac disease, and the second is psychiatric disease; 84.7% of pregnancy-related deaths are preventable [1,2]. Healthcare providers should anticipate obstetric and cardiac emergencies such as emergency cesarean delivery, postpartum hemorrhage, and peripartum arrhythmias [2], as well as preventable causes of pregnancy-related deaths such as cardiovascular disease, mental health, infection, pulmonary embolus, and injury [1].

Peripartum cardiomyopathy (PPCM), also known as postpartum cardiomyopathy, is a rare form of systolic heart failure due to left ventricular dysfunction that occurs during pregnancy or within months after delivery. Diagnostic findings often show reduced ejection fraction (EF < 45%). There may or may not be left ventricular dilatation, and it occurs without an identifiable cause, such as coronary artery disease or valvular dysfunction. Its incidence is one in 1,000-4,000 pregnancies, which increases in advanced maternal age, hypertensive disorders of pregnancy, and multiple gestations [3-5]. Data show Black and Hispanic populations and individuals with lower socioeconomic status have higher rates of dyslipidemias, hypertension, diabetes, obesity, and smoking due to barriers to cardiovascular health such as lack of insurance, high rates of unemployment, lower income, and lack of education. Cardiovascular disease or pre-existing conditions can increase the risk of developing PPCM [6]. The exact cause is not fully understood but is thought to involve a combination of factors, including nutritional deficiencies, hormonal changes, immune modulation, and genetic predisposition. These create hemodynamic and oxidative stress, leading to vascular damage of the heart. Medical management of PPCM is similar to heart failure with reduced ejection fraction (HFrEF), but adjustments are made during intrapartum to protect the fetus. PPCM can result in complete recovery, unstable arrhythmias (ventricular tachycardia (VT), ventricular fibrillation (VF)), persistent heart failure, thromboembolic events, brain injury, cardiopulmonary arrest, and death. Rapid deterioration can lead to urgent use of mechanical circulatory support and cardiac transplant [5-8].

The classical form of takotsubo cardiomyopathy (TCM), also known as stress-induced cardiomyopathy, is characterized by left ventricular dysfunction with apical ballooning without underlying coronary artery disease. Atypical takotsubo, identified in one study in up to 81.7% of cases, presents as morphologic variants, including mid-ventricular, inverted, and focal myocardial involvement [9,10]. TCM can present with symptoms similar to a heart attack with chest pain, shortness of breath, elevated troponins, and EKG findings consistent with myocardial infarction without the presence of coronary artery disease. Spontaneous recovery of cardiac function occurs within 60 days after admission for 54.9% of individuals, most of which occur prior to discharge [9]. Although the majority of patients with TCM recover, it can also lead to complications, such as ventricular fibrillation, myocardial infarction, cerebrovascular events, and death [9,11]. Emotional or psychological stressors often trigger TCM. A higher incidence occurs in patients with pre-existing psychiatric conditions, such as postpartum depression [12-16].

We present the following case of a 25-year-old female at 10 months postpartum with postpartum depression presenting with cardiac arrest and cardiomyopathy that was ultimately fatal.

## Case presentation

A 25-year-old female, para 2 gravida 2, at 10 months postpartum, arrived by EMS after becoming unresponsive at home. The patient was lying in bed when her husband noticed her breathing became irregular and then suddenly stopped. EMS initiated resuscitation efforts promptly upon arrival at their home. The patient was in ventricular fibrillation cardiac arrest and received five rounds of epinephrine and two doses of amiodarone, one at 300 mg and the second at 150 mg, which resulted in the return of spontaneous circulation (ROSC) after an unknown amount of time. En route to the ED, the patient briefly went into pulseless electrical activity (PEA), an unshockable rhythm that resolved.

**Figure 1:**
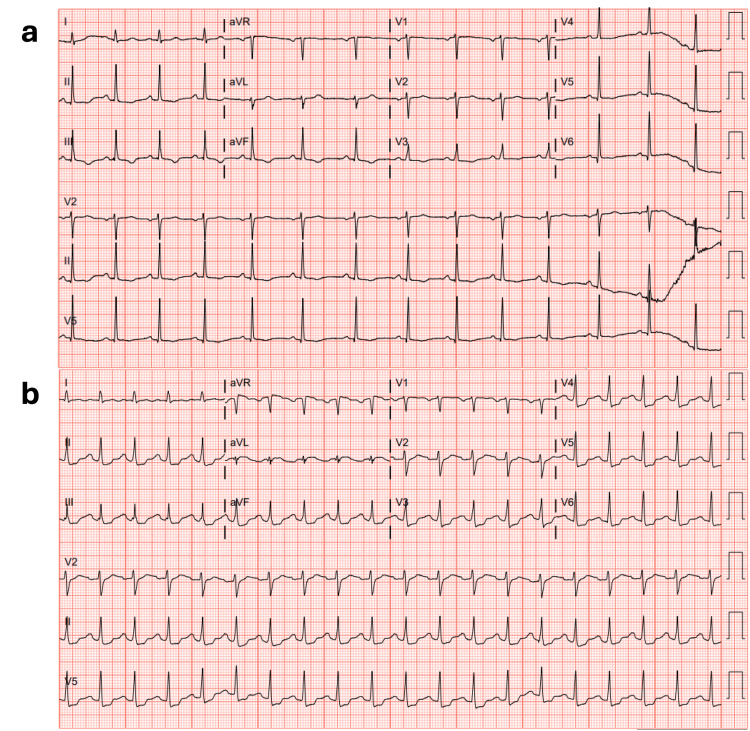
Electrocardiogram (EKG). (a) EKG from the initial ER presentation three days prior, which showed T-wave inversions in the inferior leads. There were no other EKGs for baseline comparison. (b) EKG at the second ER admission post cardiac arrest showing diffuse ST depressions with narrow QRS complexes consistent with the subendocardial injury.

On arrival, the patient had sinus tachycardia with pulses but remained unresponsive with a Glasgow Coma Scale score of 3 with no sedation or paralytic. Capillary refill was more than three seconds. The EKG on arrival showed diffuse ST depressions with narrow QRS complexes consistent with subendocardial injury. The ER physician compared it to the EKG taken three days prior, which showed T-wave inversions in the inferior leads (Figure 1). In the ED, the patient went from sinus tachycardia to ventricular tachycardia to ventricular fibrillation. The ER physician activated a code, we started CPR, and the patient received three rounds of epinephrine, one dose of magnesium, one ampule of bicarbonate, and one defibrillation at 150J, achieving ROSC. At that time, we placed an arterial line for further cardiopulmonary monitoring. To manage hypotension, we started norepinephrine, vasopressin, and epinephrine.

**Figure 2:**
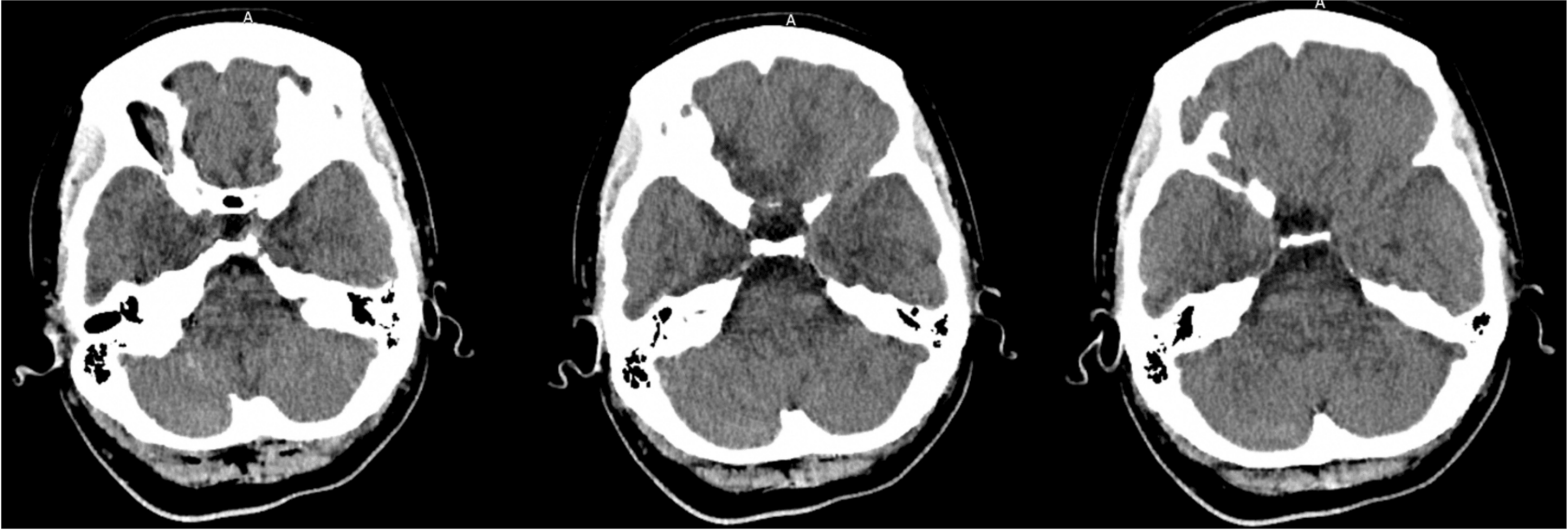
Non-contrast CT of the head. Effacement of the basal cisterns and crowding of the foramen magnum, which is likely secondary to diffuse cerebral edema. There was no significant opacification of intracranial arteries. There was opacification of external carotid artery branches.

The patient's lab values showed leukocytosis, likely due to cardiopulmonary arrest. Electrolyte levels were low, the blood glucose was high, with no history of diabetes, and elevated and upward trending troponin, indicating myocardial damage. Arterial blood gas showed a slightly elevated anion gap metabolic acidosis, likely due to lactic acidosis from post-cardiopulmonary arrest (Table 1). A post-code computed tomography angiography (CTA) of the head with and without contrast was performed. Significant findings of the CTA were effacement of the basal cisterns and crowding of the foramen magnum, which is likely secondary to diffuse cerebral edema. There was no significant opacification of intracranial arteries. There was opacification of external carotid artery branches (Figure 2). After starting permissive hypothermia, there was an additional episode of VF, which converted to sinus with a single defibrillation.

**Table 1:** Pertinent patient lab values from day one of admission and changes on day two of admission.

	Reference range	Day 1	Day 2
White blood cells (WBC)	1-4.8 × 109/L	21.4	31.5
Sodium (Na)	136-145 mEq/L	130	163
Potassium (K)	3.5-5.0 mEq/L	3	1.6
Chloride (Cl)	95-105 mmol/L	95	128
Carbon dioxide (CO2)	35-45 mm Hg	15	13
Blood urea nitrogen (BUN)	6-24 mg/dL	9	14
Creatinine	0.6-1.1 mg/dL	0.69	0.94
Troponin	≤0.04 ng/mL	183	1,364
Glucose	70-99 mg/dL	494	322
pH	7.38-7.44	7.33	
Anion gap	4-12 mEq/L	20	22
Lactic acid	0.5-2.2 mmol/L	4.5	7.7

Three days prior, the patient visited the ED for a concerning headache; she received a thorough workup of her chief concern and had a normal head CT showing no hemorrhages. The patient's blood pressure ranged from 90-113/50-58, urinalysis was negative for protein, and the only abnormality on the workup was the patient's EKG, and the interpretation stated: "ST/T wave abnormalities, considered inferior ischemia." After receiving a diagnosis of chronic migraine and being discharged, the patient went home. The EKG abnormalities should have prompted a more extensive cardiac workup. The patient’s family informed staff that the headache persisted. The patient had a history of migraines, but the headache was worse than her usual headaches. The current headache was associated with nausea and vomiting. The onset of the headache had come on like a thunderclap and was the worst headache of her life. The patient was taking Tylenol, ibuprofen, and Excedrin for the headache. She had no fevers and no evidence of subarachnoid hemorrhage on CT for the previous or current admission. The patient was also experiencing postpartum depression, but there was no concern for overdose. The patient was currently breastfeeding.

The patient arrived via flight to the neurology intensive care unit (ICU) on mechanical ventilation. We continued norepinephrine, vasopressin, and epinephrine for hemodynamic stability, and to prevent additional arrhythmias, we continued amiodarone. In the ICU, we administered high-dose steroids for refractory shock and insulin for high blood glucose. The patient was unresponsive to all stimuli, including pain, auditory, and visual stimuli. The pupils were 7 mm and not reactive to light. Additionally, the patient had no corneal, ocular, gag, or cough reflexes. We ordered a video EEG to assess brain activity and Keppra as a seizure prophylactic, with plans to get an MRI when the patient was stable enough to transfer.

**Figure 3:**
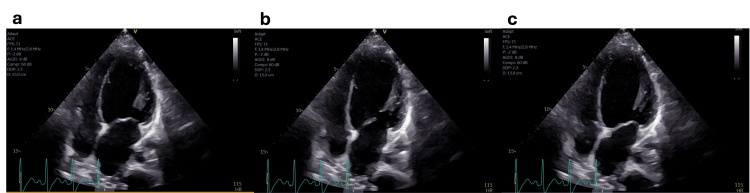
Echocardiography (ECHO). ECHO showed characteristic transient ballooning of the left ventricle, including the apex, with an ejection fraction of 20% and diffuse hypokinesis with sparing of a hyperdynamic apex. (a) Diastole before atrial kick. (b) Diastole after atrial kick. (c) Systole.

The ICU physician consulted neurology and cardiology. Neurology started mannitol and hypertonic saline for cerebral edema. Mid-America Transplant (St. Louis, Missouri) was notified about possible organ donation to be offered to the family if the patient was pronounced brain dead. Additionally, bedside echocardiography (ECHO) showed preliminary results concerning dilated cardiomyopathy, with an EF of 20%, and diffuse hypokinesis with sparing of a hyperdynamic apex (Figure 3). The ECHO was diagnostic for both postpartum cardiomyopathy and TCM shortly after she went into cardiac arrest, requiring nine minutes of resuscitation. Cardiology suspected underlying arrhythmogenic right ventricular dysplasia and recommended continuing amiodarone with an electrophysiology evaluation only if the neurological prognosis improved.

At this time, the team was suspicious of anoxic brain injury secondary to cardiac arrest. On day two of admission, neurology conducted further testing, including caloric testing, analysis of the video EEG, and a cerebral perfusion study to confirm brain death. Final radiology results proved TCM with characteristic transient ballooning of the left ventricle, including the apex and postpartum cardiomyopathy, with an EF of 20%. The EEG showed isoelectric activity, consistent with brain death. There was still no clear etiology for the patient's cardiac arrest. On the neurologic exam, the patient had an absence of all brainstem reflexes, and the ice water caloric testing was negative. There was no spontaneous breathing or sedation, and the pupils were fully dilated and nonreactive. The patient met all criteria for brain death except for the apnea test, but due to the patient's critical condition requiring multiple vasopressors, it was likely she would not survive apnea testing. The patient's family opted to defer apnea testing.

The patient's heart arrested again. At that time, the family asked to withdraw all life-saving measures, and she was shortly pronounced dead.

## Discussion

Cardiac disease is the number one cause of preventable pregnancy-related deaths in the state of Missouri. With diagnosis, monitoring, and treatment, we can decrease the risk of maternal deaths due to these conditions [1]. While the incidence of PPCM and TCM is rare, the suspicion of cardiac disease should be high during the intrapartum, peripartum, and up to 12 months postpartum. These conditions are life-threatening and should be considered a cardiac emergency, and treatment and monitoring should be initiated promptly after diagnosis [7,8,11].

Emotional or psychological stress can trigger TCM and has increased incidence in patients with premorbid psychiatric illness [12-16]. Stress and anxiety increase cortisol production. Persistently high cortisol and activation of a hyperadrenergic state can cause endothelial dysfunction. The increase of inflammatory biomarkers, interleukins, C-reactive protein, and the recruitment of macrophages causes inflammation and oxidative stress, which damages the vessel wall. There is a bi-directional relationship between mental health and cardiovascular diseases [17]. Our patient was diagnosed with postpartum depression, which increased the risk of developing TCM.

In addition, the patient was experiencing an ongoing headache that felt worse than her usual migraines occurring for three days before cardiac arrest, which could have added additional stress. The headache also could have been a warning sign. Cardiac cephalgia is a migraine-like headache that can be associated with decreased blood flow to the heart and, in some cases, can be the only presenting symptom of a heart attack. It occurs for several reasons; in our patient's case, it is more likely due to decreased venous return to the heart and increased inflammatory markers causing vasodilation [18].

Patients with PPCM can fully recover, and over half of patients with TCM alone recover within four to eight weeks. PPCM is managed with the same treatments used for HFrEF, whereas TCM is primarily supportive therapy. However, complications can arise for both diseases, such as unstable arrhythmias (VT, VF), persistent heart failure, thromboembolic events, brain injury, cardiopulmonary arrest, and death [5-9,11]. Rapid deterioration can lead to urgent use of mechanical circulatory support and cardiac transplant [5-8].

Although PPCM and TCM are rare, the risk of mortality due to cardiomyopathies in the antepartum, peripartum, and postpartum stages demonstrates the need to assess for and anticipate cardiac disease in patients even up to 12 months postpartum [4]. Our patient was approximately 10 months postpartum and presented to the ER with a headache and subtle EKG abnormalities. Due to the fact that some patients can have atypical presentations of myocardial infarction, such as cardiac cephalgia, increased workup of the EKG abnormalities should have been assessed at that time, such as cardiac enzymes, troponin, brain natriuretic peptide (BNP), and an ECHO to evaluate cardiac function. It is a class C recommendation to be aware of the signs and symptoms of cardiovascular disease, also to order baseline BNP during pregnancy, and to follow it serially throughout pregnancy and postpartum. Another class C recommendation is that all women should be screened for cardiovascular disease in the antepartum and postpartum period [18]. Our patient was diagnosed with both PPCM and TCM after experiencing complications of myocardial infarction and anoxic brain injury. The cardiomyopathies most likely presented early in her postpartum period. Patient outcomes would be impacted by increased cardiac surveillance and management in the postpartum period. According to the Centers for Medicare & Medicaid Services (CMS), echocardiography is not covered for screening purposes. However, BNP is covered for asymptomatic patients; our current recommendation for screening would involve a baseline BNP with serial measurement [19,20].

## Conclusions

Screening and managing cardiac diseases and symptoms in the peripartum and postpartum period are essential to reduce maternal mortality. Both PPCM and TCM can present with acute cardiac symptoms in the peripartum period and screening for cardiac disease should continue up to 12 months postpartum. Additionally, with significant racial disparities present regarding maternal mortality rates and increase in risk of postpartum cardiac disease established in Black and Hispanic women, continued research needs to be conducted to understand these relationships better. Healthcare providers should have a high index of suspicion for cardiac disease in pregnant women and in the postpartum period, with a low threshold for ordering EKG, BNP, and troponin tests. These markers are screening tools, and abnormal results prompt investigation with echocardiography. Our case study demonstrates a need for more evidence on the screening practices for cardiovascular disease in the peripartum, intrapartum, and postpartum periods. Current recommendations for screening and guidance are class C due to lack of evidence. We could increase the class recommendations and the likelihood of insurance coverage with more evidence. Continued efforts to establish incidence rates of TCM should be kept in mind by forensic pathologists as states adopt policies to perform autopsies on patients who have passed away within 365 days of being pregnant.
